# A systematic review of Tuina for women with primary dysmenorrhea: A protocol for systematic review and meta-analysis

**DOI:** 10.1097/MD.0000000000027935

**Published:** 2021-11-24

**Authors:** Yueming Lv, Huichao Feng, Fushi Jing, Yonghui Ren, Qian Zhuang, Jiao Rong, Qi Pan, Mengtian Li, Jing Zhang, Fujie Jing

**Affiliations:** a School of Acupuncture-Tuina, Shandong University of Traditional Chinese Medicine, Jinan, Shandong, China; b Department of Rehabilitation, The People's Hospital of Juxian, Juxian, Shandong, China; c Department of Tuina, Affiliated Hospital of Shandong University of Traditional Chinese Medicine, Jinan, Shandong, China.

**Keywords:** meta-analysis, primary dysmenorrhea, protocol, Tuina

## Abstract

**Background:**

Primary dysmenorrhea (PD) occurs most often in adolescent girls. Tuina, a kind of Chinese massage, can effectively relieve women's pain and is widely used in clinical practice. However, there is no relevant systematic review show its effectiveness and safety. The study aims to assess the effectiveness and safety of Tuina for PD.

**Methods:**

The following electronic databases will be searched from the respective dates of database inception to September 1st, 2021: The Cochrane Library, Web of Science, EMBASE, Springer, MEDLINE, China National Knowledge Infrastructure, the Chinese Biomedical Literature Database, the World Health Organization International Clinical Trials Registry Platform, the Chinese Scientific Journal Database, Wanfang database, and other sources.

**Results:**

This study will provide a high quality comprehensive and/or descriptive analysis of existing evidence on Tuina therapy for PD.

**Conclusion:**

This study will provide the evidence of whether Tuina is an effective and safe intervention for women with PD.

**PROSPERO registration number:**

CRD42021257392.

## 1 Introduction

### 1.1 Description of the condition

Primary dysmenorrhea (PD) is characterized by the presence of lower abdominal pain and distension in the lower abdomen before and after menstruation in the absence of organic lesions in the reproductive organs.^[1,2]^ The pain often lasts for 48 to 72 hours during the menstrual flow and is most severe during the first or second day of menstruation.^[3]^ Onset is typically 6 to 12 months after menarche.^[4,5]^ It can also induce other discomfort, such as nausea, vomiting, diarrhea, insomnia before or during menstruation.^[4]^ The prevalence of dysmenorrhea varied from 34% to 94%.^[6]^ The studies have shown that PD is generally associated with the following factors: smoking,^[7]^ alcohol,^[8]^ obesity,^[9]^ underweight,^[10]^ family history of dysmenorrhea,^[11]^ and skipping breakfast.^[12]^ Mental health is also linked to dysmenorrhea.^[13]^

### 1.2 Description and function of intervention

Tuina (Chinese Massage), based on the viscera theory, meridian theory, 5 elements theory, is a kind of physical therapy of traditional Chinese medicine with a long history.^[14]^ Many Tuina therapy for PD have been reported: spinal Tuina,^[15]^ viscera Tuina^[16]^ aromatherapy massage,^[17]^ rhythmical massage therapy.^[18]^ Acupressure therapy is also used to treat PD, such as pressing Sanyinjiao (SP6),^[19]^ pressing Taichong (LR3).^[20]^ The study has shown that Tuina therapy can exert pain relief effect by improving uterine blood circulation and regulating abnormal levels of PGF2a and PGE2.^[21]^

### 1.3 Why the review is important

PD occurs more often in adolescent women which is the main cause of recurrent short-term school absenteeism in adolescent girls in the United States.^[5,22]^ PD seriously affects women's life, work,^[23]^ and even mental health.^[13]^ At the same time it brings serious economic burden.^[2]^ At present, the first line of PD is NSAIDs whose adverse effects are nausea, vomiting, and heartburn.^[23]^ The clinical trials have proven that Tuina therapy is effective for dysmenorrhea.^[16,18,19]^ However, there is no literature or review to confirm the effectiveness and safety of massage therapy for PD. It is urgently needed to accomplish this review.

## 2 Methods

This systematic review has been registered in the PROSPERO network (No. CRD42021257392). All steps of this systematic review will be performed according to the Cochrane Handbook (5.2.0).

### 2.1 Selection criteria

#### 2.1.1 Types of studies

Randomized controlled trial (RCT) and blinded research will be included. Published clinical trials that reported the efficacy and safety on Tuina for women with PD will be included. RCTs that involve at least 1 Tuina related treatment to PD, and 1 control treatment (or blank treatment) will be included. As there is a risk of interference with the outcome, non-RCTs will be excluded. Studies of animal experiment, review, case report, and meta-analysis poster presentations will be excluded.

#### 2.1.2 Types of patients

The patients who were diagnosed as PD and never give birth will be included, without limits on race, nationality, and medical units.

#### 2.1.3 Types of interventions and comparisons

Interventions can be any type of Tuina: acupressure, meridian Tuina, viscera Tuina, spinal Tuina, knead ridge, and so on. Types of control interventions will be included: no treatment, placebo, and other interventions (e.g., acupuncture, cupping therapy, drugs, and physical interventions, moxibustion). However, if interventions and comparisons of the study both contain Tuina, it will be excluded. Interventions of Tuina combined with other therapies will be included, only if these combinations are compared to the other therapies semplice.

#### 2.1.4 Types of outcomes

The primary outcome will be pain relief that occurred only during the intervention according standard pain measure scale, such as visual analog scale, or numeric rating scale. Visual analog scale is scored on a 5-point-scale (0 = absent, 1 = mild, 2 = moderate, 3 = severe, 4 = very severe). The secondary outcome will include other menstrual discomfort, such as nausea, insomnia, and so on.

### 2.2 Search methods for identification of studies

#### 2.2.1 Electronic searches

The following electronic databases will be searched from the respective dates of database inception to September 1st, 2021: The Cochrane Library, Web of Science, EMBASE, Springer, MEDLINE, China National Knowledge Infrastructure, the Chinese Biomedical Literature Database, the World Health Organization International Clinical Trials Registry Platform, the Chinese Scientific Journal Database, Wanfang database, and other sources. All published RCTs about this subject will be included. Exemplary trial register of MEDLINE is listed in Table 1, terms are conformed to medical subject heading. According to the different retrieval modes, keywords may combine with free words and comprehensive search will be performed.

**Table 1:** MEDLINE search strategy.

#1 MeSH Major Topic: primary dysmenorrhea
#2 MeSH Major Topic: dysmenorrh
#3 MeSH Major Topic: menstrual disorder
#4 MeSH Major Topic: menstrual pain
#5 Title/Abstract: adolescent girls
#6 Title/Abstract: nullipara
#8 Title/Abstract: gynecologic
#9 MeSH Major Topic: Tuina
#10 MeSH Major Topic: massage
#6 Title/Abstract: nullipara #7 Title/Abstract: between the age of 14 and 28
#12 MeSH Major Topic: massotherapy
#13 MeSH Major Topic: manipulation
#14 #1 or #2 or #3 or #4
#15 #5 or #6 or #7 or #8
#16 #9 or #10 or #11 or #12 or #13
#17 #14 or #15 or #16

### 2.3 Data collection and analysis

#### 2.3.1 Selection of literature

**Figure 1:**
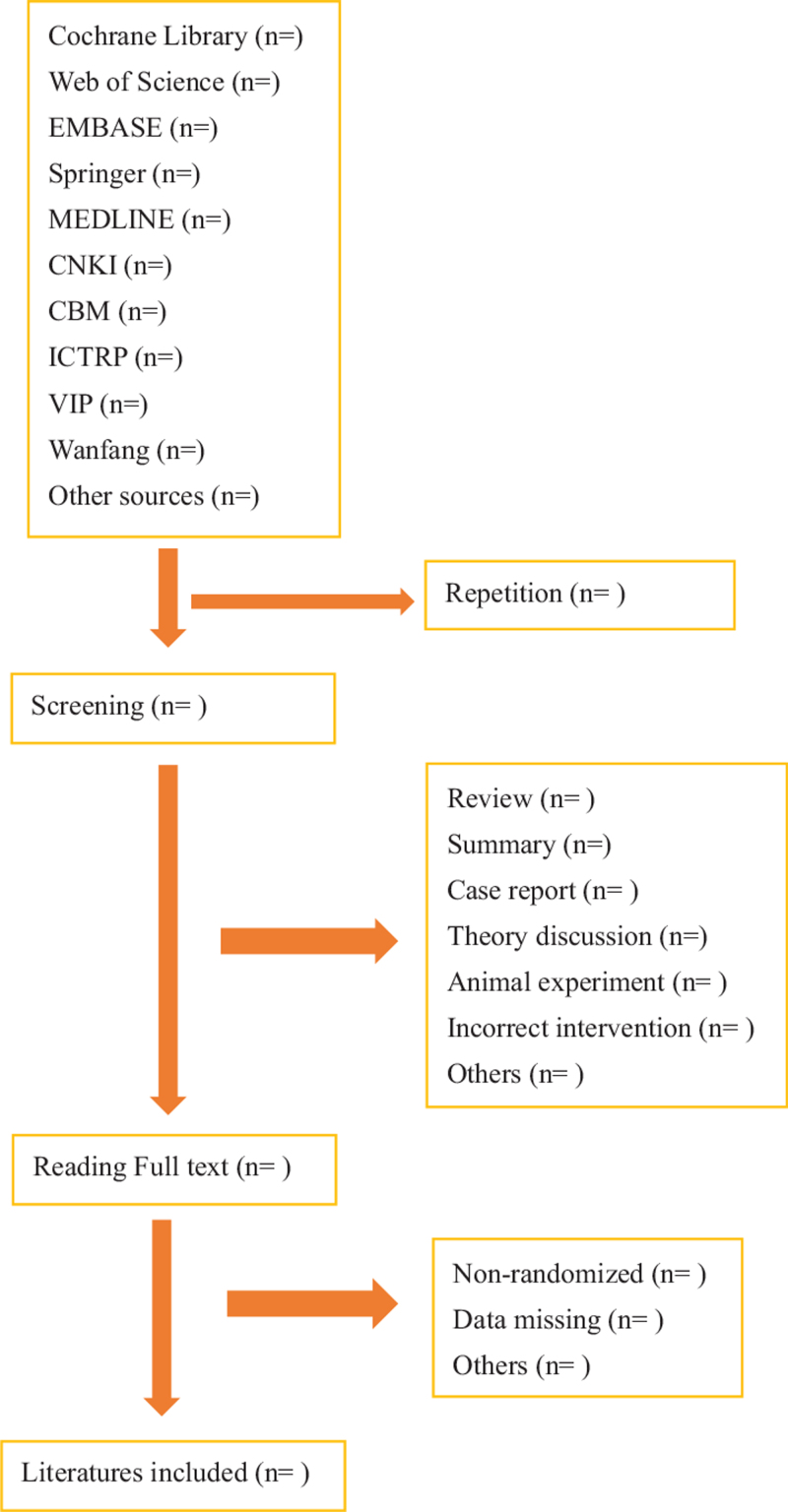
Flow diagram of literature retrieval.

Two authors (YL and HF) will select clinical trials conforming to inclusion criteria independently. After the articles are screened, the studies that are disrelated, repetitive, nonstandard will be excluded. Screening operation of the study will be outlined in Figure 1. If the full literatures are unable to obtained or related data is incomplete, we will be made to contact the corresponding author. Third-party experts will be consulted to determine the selection divergence.

#### 2.3.2 Assessment and quality of included literature

Two independent authors (YL and HF) will evaluate quality of included literature and assess the risk of bias by Review Manager (5.3.5) based on Cochrane Handbook (5.2.0). Quality will be assessed from 5 aspects: randomized method, allocation concealment, blinding methods (participants, personnel, outcome), completeness of outcome data and selective reporting. Third-party experts will be consulted to determine the selection divergence.

#### 2.3.3 Data extraction

The authors (YL and HF) plan to extract the data from the articles selected for inclusion, and to resolve differences in opinion through discussion with experts. Data will be recorded onto an electronic form, including categories for basic information about the studies (numbering, the first author's name, the year of the study was published, and the contact information for the corresponding author), the sample sizes, and grouping methods used, participant characteristics including age and gender, expressed as mean additions and subtractions above and below standard deviation and the percentages, and details of the intervention methods involved, including treatment time, the selection of acupoints, treatment efficacy, treatment cycles, side effects, and follow-up.

#### 2.3.4 Measures of treatment effect

Two authors (HF and FJ) will analyze independently and cross-check treatment effect by Review Manager 5.3.5. Continuous data will be presented by mean difference or standard mean difference with 95% confidence interval. Risk ratio with 95% CIs will be adopted if dichotomous data exists. Other binary data will be changed into the risk ratio form for analysis.

#### 2.3.5 Dealing with missing data

The authors will contact the corresponding authors by email or other contacts because of the missing essential data. If the missing data is unavailable, we will analyze the existing data and suppose the missing data as random missing.

#### 2.3.6 Assessment of heterogeneity

*Q*-test and *I*^2^ statistic with RevMan5.3.5 will evaluate the heterogeneity of studies. The studies will be deemed as low heterogeneity, if *I*^2^ the value is less than 50%; They will be considered as moderate heterogeneity while *I*^2^ the value is between 50% and 75%; and if the *I*^2^ value exceeds 75%, they are will be considered as high heterogeneity.

#### 2.3.7 Assessment of reporting bias

The plots of the funnel will be used to evaluate reporting bias and other reporting biases. Dissymmetry funnel plot means high risk of reporting bias, while the symmetric one may indicate low risk.

#### 2.3.8 Data synthesis

A meta-analysis or descriptive analysis will be carried out based on measurement methods, intervention methods, heterogeneity levels, among others. If clinical and methodological heterogeneity are low, the fixed-effect model will be applied by merger analysis; the random-effects model will be applied by merger analysis when heterogeneity indicates a moderate level. If a significant level of heterogeneity is found, a descriptive analysis will be performed instead.

#### 2.3.9 Subgroup analysis

Subgroup analysis will be performed based on the findings from the data synthesis. If the heterogeneity is found to have been caused by particular features of the included studies (e.g., the intervention methods, such as type, time, and cycle, and the measurement methods used in the clinical trials), subgroup analysis will be conducted relevant to these categories.

## 3 Discussion

The incidence of PD is high and it seriously affects women's quality of life. As a safe and effective treatment, Tuina is widely used in the treatment of PD in clinical. In recent years, more and more clinical cases of Tuina therapy for PD have been reported. However, high quality trail is still insufficient. This review will begin when necessary trails are meeting. In order to give compelling evidence and better guide in clinic practice, all actions of this review will be performed according to Cochrane Handbook 5.2.0.

## Author contributions

**Conceptualization:** Yueming Lv, Huichao Feng.

**Data curation:** Fushi Jing, Yonghui Ren, Mengtian Li.

**Investigation:** Jing Zhang, Qian Zhuang, Jiao Rong.

**Methodology:** Yueming Lv.

**Supervision:** Huichao Feng, Qi Pan.

**Visualization:** Yueming Lv, Fujie Jing.

**Writing – original draft:** Yueming Lv, Huichao Feng.

**Writing – review & editing:** Fujie Jing.
